# IL-37 Confers Anti-Tumor Activity by Regulation of m6A Methylation

**DOI:** 10.3389/fonc.2020.526866

**Published:** 2021-01-08

**Authors:** Xiaofeng Mu, Qi Zhao, Wen Chen, Yuxiang Zhao, Qing Yan, Rui Peng, Jie Zhu, Chunrui Yang, Ketao Lan, Xiaosong Gu, Ye Wang

**Affiliations:** ^1^Academy of Medical Engineering and Translational Medicine, Tianjin University, Tianjin, China; ^2^Clinical Laboratory, Qingdao Central Hospital, The Second Affiliated Hospital of Medical College of Qingdao University, Qingdao, China; ^3^Department of Hyperbaric Oxygen, Qingdao Central Hospital, The Second Affiliated Hospital of Medical College of Qingdao University, Qingdao, China; ^4^Institute of Bioengineering, Biotrans Technology Co., LTD., Shanghai, China; ^5^United New Drug Research and Development Center, Biotrans Technology Co., LTD., Ningbo, China; ^6^Department of Pathology, Second Hospital of Tianjin Medical University, Tianjin, China

**Keywords:** N6-methyladenosine, RNA methylation, interleukin 37, lung cancer, A549 cells

## Abstract

N6-methyladenosine (m6A) is a common transcriptomic modification in cancer. Recently, it has been found to be involved in the regulation of non-small cell lung cancer (NSCLC) formation and metastasis. Interleukin 37 (IL-37) plays a crucial protective role in lung cancer. In our previous studies, we found that IL-37 is a potential novel tumor suppressor by inhibiting IL-6 expression to suppress STAT3 activation and decreasing epithelial-to-mesenchymal transition. Moreover, we found that treatment of IL-37 in lung cancer cells induced widespread and dynamic RNA m6A methylation. The effects of RNA m6A methylation of IL-37 treatment require further study. However, the functions of RNA m6A methylation of IL-37 treatment still await elucidation. Using MeRIP-seq and RNA-seq, we uncovered a unique m6A methylation profile in the treatment of IL-37 on the A549 cell line. We also showed the expression of m6A writers METTL3, METTL14, and WTAP and erasers ALKBH5 and FTO in A549 cells and lung cancer tissues after the treatment of IL-37. This study showed that IL-37 could lead to changes in m6A methylation level and related molecule expression level in A546 cells and may downregulate the proliferation by inhibiting Wnt5a/5b pathway in A549 cells. We conclude that IL-37 suppresses tumor growth through regulation of RNA m6A methylation in lung cancer cells.

## Introduction

N6-methyladenosine (m6A) is a common RNA modification and has been shown to be critically important in the regulation of tumorigenesis of alternative splicing, stability, and translation (1). m6A profiling experiments in various species revealed that its enrichment not only in ribosome-associated mRNA but also near stop codons or long internal exons (2–4). Regulation by m6A is determined by m6A methyltransferases (writers, including METTL3/4/14) (5), m6A-binding proteins [readers, including fat mass and obesity-associated protein (FTO), and AlkB homolog 5 (ALKBH5)] (6, 7) and m6A demethylases (erasers, including YTHDC1 and YTHDF1) (8, 9). The role of m6A methylation is essential in various important biological processes, such as cellular differentiation, pluripotency, and stress response (10–12), particularly in cancer stem cell self-renewal and differentiation. Therefore, m6A may serve as an important pathway regulating initiation and progression in cancers (13, 14).

Interleukin (IL)-37 has been reported to have antitumor effects in hepatocellular carcinoma (HCC) (15), fibrosarcoma (16), breast cancer (17), and so on. IL-37 isolated *in silico* in 2000 (18), belongs to the IL-1 family and is also named IL-1F7. The IL-37 gene was mapped to chromosome 2, and the six exons of the IL-37 gene encode five isoforms (IL-37a–e). IL-37b is the largest isoform and has been best characterized so far (19). The N-terminus of IL-37 encloses a caspase-1 cleavage site (20) and must be cleaved by caspase-1 to be activated (21). IL-37 acts as an anti-inflammatory cytokine by inhibiting innate responses (22) and plays a pivotal role in acute and chronic inflammation inflammation by balancing the cytokine expression (23). Therefore, IL-37 may serve as a potential key factor in restoring inflammatory balance in cancer development and treatment. In our previous study, we found IL-37 can inhibit cell invasion and metastasis through the IL-6/STAT3 signaling in non-small cell lung cancer (NSCLC) (24). However, how IL-37 affects cell growth and its underlying mechanisms have not been fully elucidated.

How IL-37 suppresses human lung adenocarcinoma growth is not completely understood. Given broad regulatory roles of IL-37 in cell growth, we hypothesized that IL-37 confers anti-tumor activity through regulation of m6A methylation. We studied the m6A status of IL-37–treated human lung adenocarcinoma A549 cells and investigated the differences between the m6A modification patterns of untreated controls and the IL-37 treated model. To explore the protective role of IL-37 in lung cancer, we performed an m6A-specific RNA immunoprecipitation assay coupled with high throughput sequencing (MeRIP-seq). We also tested the expression of m6A writers METTL3, METTL14, and WTAP and of erasers ALKBH5 and FTO after the treatment of IL-37 in A549 cells and lung cancer tissues. This study showed that IL-37 could lead to changes in m6A methylation level and related molecule expression level in A546 cells, and may downregulate the proliferation by inhibiting Wnt5a/5b pathway in A549 cells.

## Material and Methods

### Cell Culture and Treatment

The human NSCLC A549 cell line was obtained from the Shanghai Institute of Cell Biology of the Chinese Academy of Sciences (Shanghai, China) and preserved in the biotechnology therapeutic center at our hospital. Cells were cultured at 37°C in 90% Dulbecco’s modified Eagle medium (DMEM) (Thermo Fisher Scientific, Inc., Waltham, MA, USA) plus 10% fetal bovine serum (FBS) (Life Technologies, Gaithersburg, MD, USA) supplemented with 1% penicillin/streptomycin. Cells were treated with lentivirus (Lv)-expressing IL-37 (HanBio, Shanghai, China) for at least 72 h. Lv-expressing green fluorescent protein (GFP) served as a control.

### Global m6A/m Measurements

The global m6A/m in total RNA was detected with the EpiQuik m6A/m RNA Methylation Quantification Kit (Epigentek Group, Farmingdale, NY, USA) following manufacturers’ specifications with 100–300 ng input RNA (in triplicate).

### RNA Preparation, RNA MeRIP-Seq Library Construction and Sequencing

For Lv-IL-37 or Lv-GFP treated cells, three biological replicates were selected. Total RNA was extracted from A549 cells using an RNeasy^®^ Mini Kit (QIAGEN, Valencia, CA, USA) according to the manufacturer’s directions. To eliminate the ribosomal RNA from total RNAs, we used the Ribo-Zero rRNA Removal Kit (Illumina, Inc., CA, USA), and the RNA was then fragmented into pieces of about 100 nt nucleotides using the M220 Focused-ultrasonicator (Covaris. Woburn, MA, USA). Fragmented RNA was incubated with anti-m6A antibody 202,003 (Synaptic Systems, Göttingen, Germany) for 2 h in IP buffer (50 mM Tris-HCl, 750 mM NaCl and 0.5% Igepal CA-630) in accordance with a previously referenced study (25). The mixture was then purified with Protein A beads and precipitated by 75% ethanol. Purified RNA was used for NEBNext^®^ Ultra™ Directional RNA Library Prep Kit (New England Biolabs, MA, USA) according to a published protocol (26). Sequencing was performed on an Illumina HiSeq 4000 sequencer (Illumina, Inc.) with 2 × 100 100 cycles Solexa paired-end sequencing.

### Data Analysis

Sequence analyses were performed using the procedure described by Luo, Zhang et al. (27). The Q30 was used as quality control of the paired-end reads, the 3′ adaptors were trimmed, and cutadapt software (v1.9.3) was used to remove the low-quality reads. HISAT2 software (v2.0.4) was used to align the clean reads of all libraries to the reference genome (hg18) (28). MACS software was used to detect the peaks with a score (−10*log10, *p*-value) of >3 (29). Differentially methylated sites with a fold change cutoff of ≥2 and a false discovery rate cutoff of ≤0.0001 were identified using the diffReps differential analysis package (30). Gene expression was calculated with Cufflinks (31) using the input-sequencing reads, and Cuffdiff software was used to find the different expression genes of IL-37 treated and untreated cells. DAVID tool and Kyoto Encyclopedia of Genes and Genomes (KEGG) were used for gene function analysis (GO enrichment) pathway enrichment analysis. The *p*-value denotes significant pathway correlated to the conditions.

### Western Blotting

Lv-IL-37 treated and untreated A549 cells were collected and ruptured with RIPA lysis buffer (Sigma-Aldrich, St. Louis, MO, USA) containing 5 mM of EDTA, PMSF, cocktail inhibitor, and phosphatase inhibitor cocktail. Cell extracts were resolved by SDS-polyacrylamide gel electrophoresis and transferred onto polyvinylidene difluoride (PVDF) membranes. Membranes were blocked with 5% BSA in tris-buffered saline containing 0.1% Tween 20 (TBST buffer) and incubated with anti-Wnt5a antibody (ab179824) and anti-Wnt5b antibody (ab124818, Cambridge, UK). The membranes were then incubated with secondary antibodies and appropriate chemiluminescent substrates.

### Proliferation Assay

Lv-IL-37 treated and untreated A549 cells (5 × 103) were seeded into 96 well plates, and proliferation assay was performed with the MTT (3-(4, 5-dimethyl thiazol-2-yl) 2, 5-diphenyltetrazolium bromide) method. Four, 24, 48, 72, and 96 h later, 10 μl MTT (final concentration, 5 mg/ml) was added, and the purple formazan crystals were then dissolved by adding 100 μl acid-isopropanol (0.04 N HCl in isopropanol) into each well. The absorbance was determined at 550 nm against a reference wavelength of 630 nm and stimulation index (SI).

### Transwell Cell Migration and Invasion Assay

Transwell cell migration and invasion assay were analyzed according to the procedure described by Justus CR (32).

### Statistical Analysis

Two-tailed *t*-test was used to identify significantly different expression level between two groups (p < =0.05) in western blotting. Data are presented as the mean ± SEM.

## Results

### Expression of m6A Methylation Related Proteins in IL-37 Treated A549 Cells

**Figure 1:**
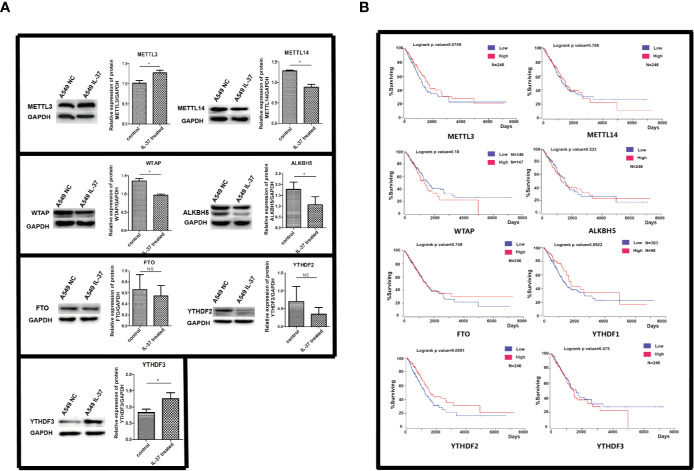
**(A)** Expression of m6A methylation related proteins in IL-37 treated A549 cells. Data are mean ± SEM. *P < 0.05. **(B)** The results of survival analysis of proteins with m6A changes using public lung cancer data from TCGA database.

Firstly, we tested the expression of methylation related proteins by western blot. As shown in **Figure 1A**, the protein level of METTL3 and YTHDF3 were much higher in IL-37 treated A549 cells than in non-treated control. The protein expression of METTL14, WTAP, and ALKBH5 decreased in IL-37 treated A549 cells, compared with that in non-treated control. However, there were no significant differences of FTO and YTHDF2 between IL-37 treated A549 cells and control. From these results, the downregulation of ALKBH5 maybe one of the potential reasons for the significant decrease in the overall methylation of RNA after IL-37 treatment of A549 cells. As shown in **Figure 1B**, survival analysis of functional genes related to m6A modification shows that YTHDF2 significantly affects the survival of lung cancer patients, and the low expression of YTHDF2 is a visible risk factor, which indicates that the expression level of m6A reader is related to the prognosis of lung cancer patients.

### The Expression of IL-37 Is Downregulated in Human Lung Adenocarcinoma

**Figure 2:**
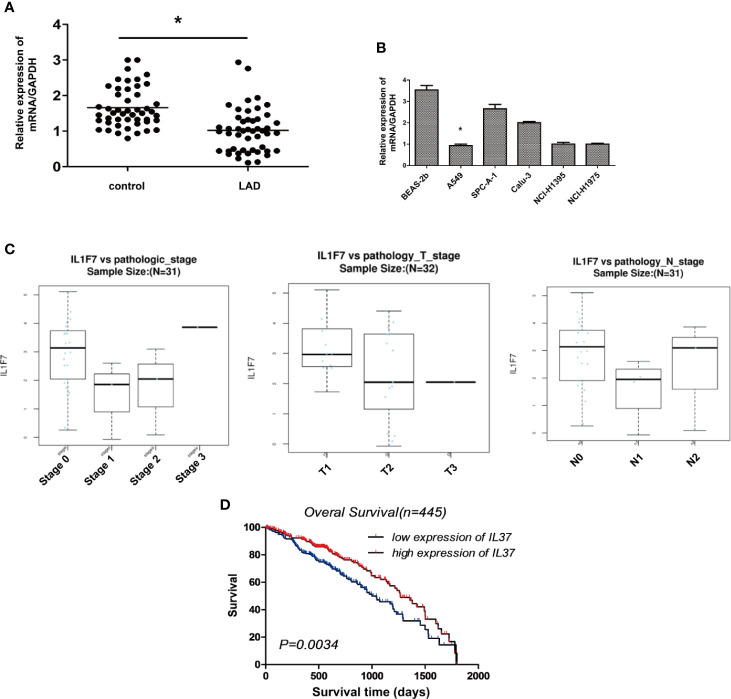
The expression of IL-37 is downregulated in human lung adenocarcinoma (LAD). **(A)** The results of differential expression analysis of IL-37 between the lung adenocarcinoma and matched tumor normal tissues. Data are mean ± SEM (n = 48 per group); **(B)** The expression of IL-37 in different LAD cell lines. Data are mean ± SEM (n = 6 per group); **(C)** The degree of IL-37 downregulation was greater in stages I and II than in stages 0 in primary LAC tumors. Data are mean ± SEM (n = 10 per group); **(D)** Results of analysis from starBase. Data are mean ± SEM. *P < 0.05.

Data on mRNA expression from lung adenocarcinoma and matched adjacent, non-cancerous tissues were downloaded from the Cancer Genome Atlas (TCGA) data portal (https://tcga-data.nci.nih.gov/tcga/) that represent a total of 48 lung adenocarcinoma samples without metastases. The differential expression analysis of IL-37 between the lung adenocarcinoma and matched tumor normal tissues showed that the expression of IL-37 was lower in lung adenocarcinoma tissues than in adjacent, non-cancerous tissues (**Figure 2A**). Compared with normal human lung epithelial cell line BEAS-2b, a similar downregulation of IL-37 was also observed in human LAD A549, SPC-A-1, Calu-3, NCI-H1395, NCI-H1975 cell lines (**Figure 2B**). In primary tumors, the degree of IL-37 downregulation was greater in stages I and II than in stages 0 (**Figure 2C**). Based on the data of 445 cases from cbioportal (https://www.cbioportal.org/), and the analysis of Kaplan–Meier (http://kmplot.com/analysis/), IL-37 might have an effect on overall patient survival, which suggesting IL-37 can be potentially used as an independent prognostic marker (**Figure 2D**).

### General Features of m6A Methylation in IL-37 Treated and Untreated A549 Cells

**Figure 3:**
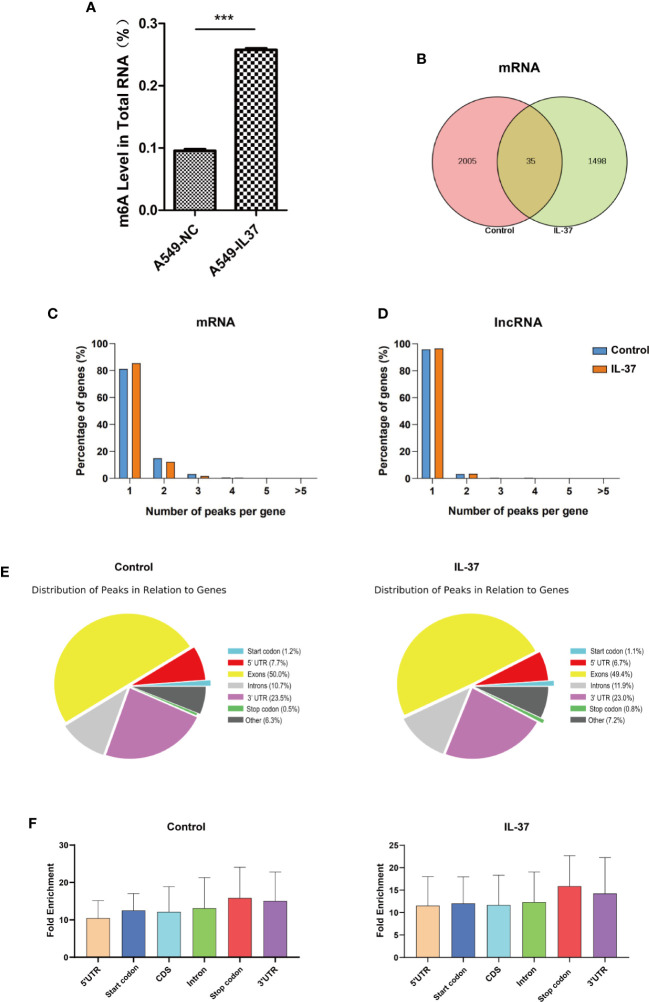
General features of m6A methylation in IL-37 treated and untreated A549 cells. **(A)** Higher global m6A modification sites in IL-37 treated cells than in IL-37 untreated cells. Data are mean ± SEM (n = 6 per group) (*** means *P* < 0.001); **(B)** Venn diagram showing the overlap of m6A peaks within mRNAs in two groups; **(C)** Proportion of mRNAs harboring different numbers of m6A peaks in two groups. The majority of genes harboring only one m6A peak; **(D)** Proportion of lncRNAs harboring different numbers of m6A peaks in two groups. The majority of genes harboring also only one m6A peak; **(E)** Pie charts showing the percentage of m6A peaks in nonoverlapping segments of transcripts; **(F)** Distributions of fold enrichment of m6A peaks. Data are mean ± SEM.

To identify the role of IL-37 on the alteration of m6A modification in A549 cells, we compared the m6A distribution in control and IL-37 treated cells using the colorimetric m6A quantification assay. We found higher global m6A modification sites in IL-37 treated cells than in IL-37 untreated cells (**Figure 3A**).

To explore the mechanism underlying IL-37’s inhibition of cell invasion and metastasis through alteration of m6A modification, we mapped the m6A methylome in IL-37 treated and untreated A549 cells by methylated RNA immunoprecipitation coupled with next generation sequencing (MeRIP-seq or m6A-seq). The results revealed significantly different m6A methylome profiles. There were 2005 non-overlapping m6A peaks in IL-37 untreated cells within 1647 coding gene transcripts (mRNAs) and 231 non-overlapping m6A peaks within 220 long non-coding RNAs (lncRNAs) in two biological replicates. In IL-37 treated cells, there were 1,498 non-overlapping m6A peaks within 1,300 mRNAs and 180 non-overlapping m6A peaks within 175 lncRNAs in two biological replicates. Of these, 35 peaks within mRNAs were overlapped between the IL-37 treated and untreated cells (**Figure 3B**) and only one peak within lncRNAs was overlapped (**Figure S1**).

The results of the motif analysis of 1,000 peaks within mRNAs with the highest scores (−10*log10, p-value) showed that IL-37 binding sites have abundant conserved m6A motif (**Figure 3C**).

We also found that, for mRNA assay, 81.2% of the m6A-methylated coding genes in the control group (85.5% in the IL-37 treated cells) contained only one m6A peak, and 14.9% of the m6A-methylated coding genes in the control group (12.2% in the IL-37 treated cells) contained two m6A peaks (**Figure 2D**). For lncRNA assay, 96.0% of the m6A-methylated coding genes in the control group (96.6% in the IL-37 treated cells) contained only one m6A peak, and 3.19% of the m6A-methylated coding genes in the control group (3.43% in the IL-37 treated cells) contained two m6A peaks (**Figure 3D**).

We further investigated the m6A distribution patterns of total peaks within mRNAs. Peaks were categorized into seven transcript segments: the 5′ untranslated region (UTR), the start codon segment (400 nucleotides centered on the start codon), the stop codon segment (400 nucleotides centered on the stop codon), the 3′ UTR, the intron region, the exon region, and the unknown region (other). The results show a similar pattern of total m6A distribution in the control and in IL-37 treated cells. In the control group, the total m6A peaks included 1,021 peaks from exon (50.05%), 480 peaks from 3′ UTR (23.53%), 218 peaks from intron (10.69%), and 158 peaks from 5′ UTR (7.75%). In IL-37 treated cells, the total m6A peaks included 758 peaks from exon (49.45%), 352 peaks from 3 UTR (22.96%), 182 peaks from intron (11.87%), and 102 peaks from 5– UTR (6.65%) (**Figure 3E**). We also found that m6A peaks had a higher fold enrichment in stop codon segments both in the control group and IL-37 treated cells (**Figure 3F**). The profiling of the m6A peaks or signals compared between the IL-37 and control samples is shown in **Tables S1** and **S2**.

### M6A Distribution of Differentially Methylated m6A Sites

We next analyzed the distribution of m6A for both IL-37 treated and untreated cells. In total, we identified 604 differentially hypermethylated sites within 567 coding genes and 451 differentially hypomethylated sites within 430 coding genes (IL-37 treated cells *vs* untreated cells) (**Table 1**). Also, 198 DMMSs within 192 lncRNA genes were hypermethylated sites, and 165 DMMSs within 160 lncRNA genes were hypomethylated sites (IL-37 treated cells *vs* untreated cells) (**Table 1**). **Tables 2** and **3** show the top ten hypomethylated m6A sites within mRNAs with the highest fold change values.

**Table 1:** General numbers of differentially methylated peaks and associated genes.

Item	Upmethylated peak	Upmethylated gene	Downmethylated peak	Downmethylated gene
mRNA	604	567	451	430
lncRNA	198	192	165	160

**Table 2:** Top 10 upmethylated peaks.

Chromosome	txStart	txEnd	Gene name	Fold change
chr2	131414301	131414500	POTEJ	117.5
chr21	39671701	39671920	KCNJ15	93.1
chr19	54720201	54720500	LILRB3	74.7
chr1	53074221	53074420	GPX7	73.7
chr4	73148821	73149120	ADAMTS3	70.9
chr6	117710661	117710860	ROS1	66.5
chr11	64513981	64514180	PYGM	53.9
chr18	65177321	65177540	DSEL	50.3
chr19	46916541	46916780	CCDC8	49.1
chr1	31887221	31887420	SERINC2	49.1

txStart/txEnd, Start/end position of the differentially methylated RNA peaks.

**Table 3:** Top 10 downmethylated peaks.

Chromosome	txStart	txEnd	Gene name	Fold change
chr2	231281470	231281596	SP100	103.2
chr3	156763561	156763780	LEKR1	84.1
chr2	207041181	207041460	GPR1	81.6
chr9	684421	684574	KANK1	75.3
chr3	52715761	52715836	PBRM1	48.3
chr16	3293321	3293540	MEFV	48.3
chr11	129733530	129733640	NFRKB	47.1
chr21	30925961	30926025	GRIK1	45.8
chr2	61404552	61404560	AHSA2	45.8
chr9	113312128	113312340	SVEP1	44.5

txStart/txEnd, Start/end position of the differentially methylated RNA peaks.

To access the distribution profile, we mapped all DMMSs within mRNAs and lncRNAs to chromosomes, as shown in –When the number of DMMSs harbored by chromosomes was normalized by the length of the respective chromosomes, the altered m6A peaks were transcribed from chromosomes 19, 17, 16, and 12 (**Figure S2B**). Further analysis showed that most identified DMMSs within mRNAs were mainly enriched in coding sequence (CDS) (**Figure S2C**). Of both the hypermethylated and hypomethylated sites, those within the 3′ UTR had the highest fold change (**Figure S2D**).

### GO and KEGG Pathway Analysis

**Figure 4:**
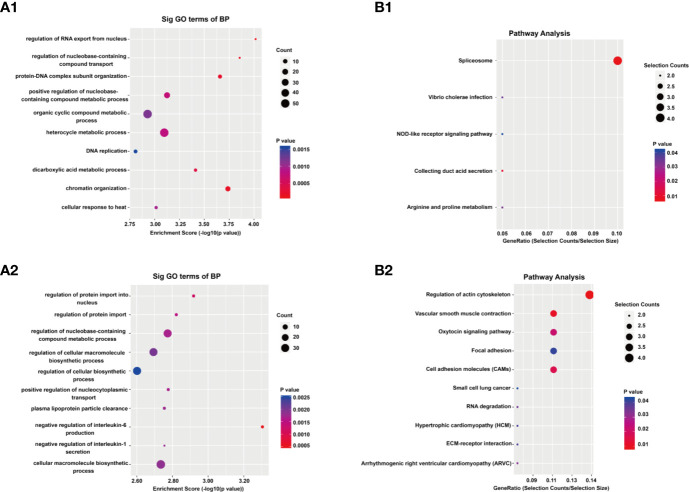
Results of Gene ontology (GO) and Kyoto Encyclopedia of Genes and Genomes analyses. **(A)** Gene ontology (GO) analyses of coding genes harboring differentially methylated N6-methyladenosine sites. The GO classifications biological process (BP) in the most significantly hypermethylated genes (A1); BP in the most significantly hypomethylated genes (A2). **(B)** Kyoto Encyclopedia of Genes and Genomes analyses of coding genes harboring differentially methylated N6-methyladenosine sites. Pathway analysis of the most significantly hypermethylated genes (B1); Pathway analysis of the most significantly hypomethylated genes (B2).

**Figure 5:**
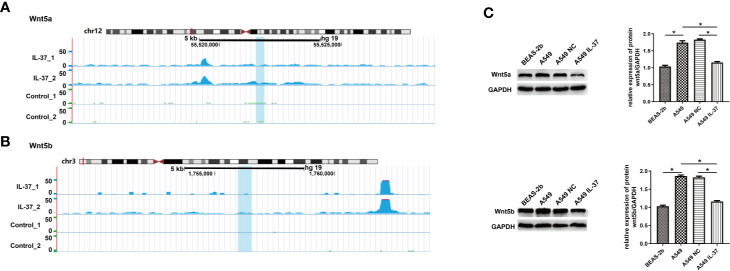
Differentially methylated RNAs are involved in Wnt biological pathway of IL-37 treated A549 cells. **(A)** Wnt-5a, within which m6A was hypermethylated (IL-37 treated A549 cells vs untreated control) near the start codon; **(B)** Wnt-5b, within which m6A was hypomethylated upstream of the 5′ UTR; **(C)** Expression of Wnt5a/5b in IL-37 treated A549 cells. Data are mean ± SEM. *P < 0.05.

To reveal the functions of m6A methylation in IL-37 treated A549 cells, protein coding genes containing DMMSs were tested by GO and KEGG pathway analysis. For the biological process (BP) category, genes with up-methylated m6A sites were significantly (p < 0.05) enriched in regulation of RNA exported from the nucleus, nucleobase-containing compound transports, protein–DNA complex subunit organization, and positive regulation of nucleobase-containing compound metabolic process (**Figure 4A1**), while genes with down-methylated m6A sites were highly enriched in regulation of protein imported into the nucleus, regulation of protein imports, regulation of nucleobase-containing compound metabolic process, and regulation of cellular macromolecule biosynthetic process (**Figure 4A2**). The results of the cellular component (CC) and molecular function (MF) are shown in **Figure S3**. The results of the KEGG pathway analysis of DMMS- containing lncRNA-associated genes demonstrated that hypermethylated genes were significantly associated with regulation of the NOD-like receptor signaling pathway and arginine and proline metabolism (**Figure 5A**). Hypomethylated genes were significantly associated with regulation of the actin cytoskeleton pathway, oxytocin signaling pathway, and cell adhesion molecules (CAMs) pathway (**Figures 4B1, 4B2**). These results suggest that differentially methylated RNAs are involved in important biological pathways of IL-37 treated A549 cells, such as Wnt-5a, within which m6A was hypermethylated (IL-37 treated A549 cells *vs* untreated control) near the start codon (**Figure 5A**), and Wnt-5b, within which m6A was hypomethylated upstream of the 5′ UTR (**Figure 5B**). Next, the protein expression of Wnt5a and Wnt5b was also down-regulated after IL-37 treatment (**Figure 5C**). Whereas, the correlation between down-regulation of ALKBH5 and Wnt5a or Wnt5b should be investigated further.

### Analysis of RBPs of Differentially Methylated mRNAs

**Figure 6:**
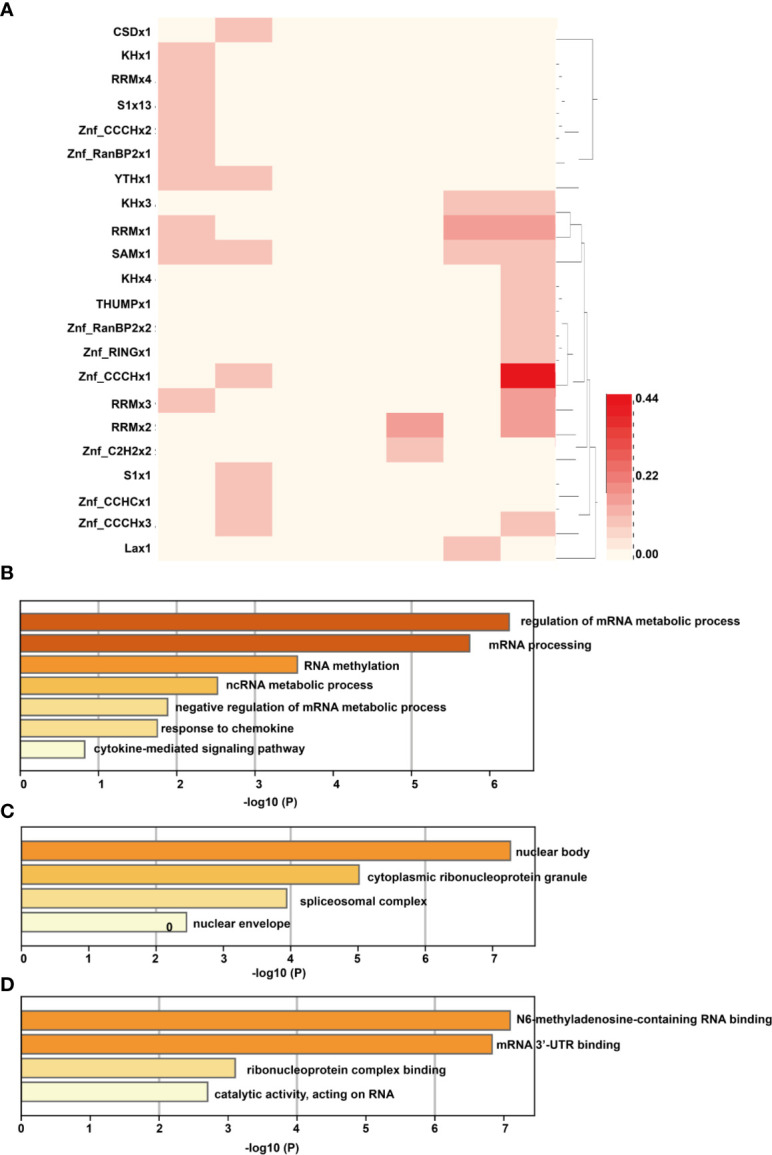
Analysis of RBPs of differentially methylated mRNAs. **(A)** The RNA binding proteins of differentially methylated mRNAs; the column of the heatmap represents the expression level of RNA (log2FC), with the first column on the left being −3 and the last column on the right as 3. **(B)** For the BP category, RBP genes were mainly enriched in processes associated with regulation of the mRNA metabolic process and cytokine-mediated signaling pathway; **(C)** For the CC category, RBP genes were enriched in the cytoplasmic ribonucleoprotein granule; **(D)** For the MF category, those genes were mainly related to N6-methyladenosine-containing RNA binding, mRNA 3′ UTR binding, and ribonucleoprotein complex binding.

To investigate how the above DMMSs act on the genes, we analyzed the RBPs of differentially methylated mRNAs through the RMBase v2.0 database (33). From this database, we obtained the modification sites and related RBP-binding regions. A total of 22 proteins were predicted to be RBPs (**Figure 6A**). The RBPs were highly distributed with a fold change (log2) = approximately −2. Then, GO enrichment analysis was performed to determine the function of the RBP genes. For the BP category, RBP genes were mainly enriched in processes associated with regulation of the mRNA metabolic process and cytokine-mediated signaling pathway (**Figure 6B**). For the CC category, RBP genes were enriched in the cytoplasmic ribonucleoprotein granule (**Figure 6C**). For the MF category, those genes were mainly related to N6-methyladenosine-containing RNA binding, mRNA 3′ UTR binding, and ribonucleoprotein complex binding (**Figure 6D**).

## Discussion

In the tumor microenvironment, production and secretion of multiple cytokines are disordered, and the immune function of the body is dysfunctional, which reduces the body’s anti-tumor ability (34, 35). In recent years, changes in the expression levels of various cytokines have become a hot topic in tumor immunology research.

IL-37 is not only an inflammatory inhibitor but also an inhibitor of inherent inflammatory and immune responses (36), and it may play a role in inhibiting tumor growth in the tumor microenvironment (37). It was reported the elevated serum levels of interleukin-37 was correlated with poor prognosis in gastric cancer (38). The level of IL-37 in the serum of renal cell carcinoma patients was significantly lower than that in a healthy control group and was negatively correlated with the TNM Classification of Malignant Tumors (TNM) stage of the tumor (39). When IL-37 levels are normal, non-hepatocellular carcinoma tissues are minimal and negatively correlated with the tumor size, microvascular metastasis, and BCLC staging of hepatocellular carcinoma (40). Our previous research showed the plasma IL-37 level in an NSCLC group was significantly lower than that in a healthy control group and that a more advanced TNM stage was associated with a more precipitous drop in IL-37 (24). We found that a decrease in IL-37 expression level is closely related to the occurrence and development of NSCLC and that increased tumor malignancy is associated with a more significant decrease in IL-37 expression. In this study, we found that the expression of IL-37 in lung adenocarcinoma tissue was significantly reduced compared to adjacent controls. After IL-37 was overexpressed in lung cancer cell line A549 cells, the overall methylation level of RNA increased significantly, suggesting that the inhibitory effect of IL-37 on the proliferation of lung adenocarcinoma cells may be caused by RNA methylation.

The most common RNA methylation modifications are m6A (N6-methyladenosine, 6-methyl adenine) and uridylation (U-tail) (25). m6A modification occurs in the methylation modification of the adenine (A) of RNA, such as mRNA and lncRNA, and U-tail occurs in the uracil modification of RNA, which is usually related to the RNA degradation. RNA modification can regulate the stability, localization, transport, splicing, and translation of RNA at the post-transcriptional level, such as the translation and alternative splicing of mRNA and the maturation of microRNA (41). Reversible RNA methylation regulates gene expression mainly at the post-transcriptional level.

Before its identification as an RNA demethylase, ALKBH5 is a dioxygenase that uses *α*-ketoglutarate and O2 as substrates in the m6A demethylation reaction (7). It was reported that hypoxia induces the breast cancer stem cell phenotype by HIF-dependent and ALKBH5-mediated m6A-demethylation of NANOG mRNA (42). ALKBH5 also maintains tumorigenicity of glioblastoma stem-like cells by sustaining FOXM1 expression and cell proliferation program (14). In the present study, we found the expression of ALKDH5 decreased significantly after IL-37 overexpression, which is potentially a reason for the significant decrease in the overall methylation of RNA.

The Wnt signaling pathways are a group of signal transduction pathways which begin with proteins that pass signals into a cell through cell surface receptors (43). It directly controls the expression levels of a large number of genes related to growth and metabolism and is involved in the regulation of a variety of biological processes, including embryonic growth and morphological development, tissue stability, the balance of energy metabolism, and stem cell maintenance. The excessive activation of the Wnt pathway is closely related to the occurrence of a variety of cancers (including colon cancer, gastric cancer, and breast cancer) (44). In this study, we found the expression of Wnt5a/5b decreased significantly, which prompted us to speculate that Wnt5a/5b may be affected by IL-37 methylation. However, the mechanisms of how IL-37 affected Wnt5a/5b pathway need to be further studied.

## Data Availability Statement

The original contributions presented in the study are publicly available. This data can be found here: https://www.ncbi.nlm.nih.gov/sra/PRJNA603214.

## Ethics Statement

This study was approved by the Institutional Ethics Committee for Clinical Research and Animal Trials of the Qingdao Central Hospital, The Second Affiliated Hospital of Medical College of Qingdao University. Informed consents were obtained from all patients before analysis. All the experimental methods involved in this study comply with the Helsinki Declaration.

## Author Contributions

XM, QZ, WC, and RP conceived and performed experiments. YZ and QY analyzed data and performed the bioinformatics analysis. LX, JL, JZ, and CY performed experiments. KL and XG provided supervision. YW conceived the project, analyzed data, wrote the manuscript, and provided supervision. All authors contributed to the article and approved the submitted version.

## Funding

This study was supported by the National Natural Science Foundation of China (No. 81670822, 81370990, and 81800805), and Qingdao Key Research Project (No. 17-3-3-10-nsh and 19-6-1-3-nsh).

## Conflict of Interest

Author YZ was employed by Biotrans Technology Co., LTD.

The remaining authors declare that the research was conducted in the absence of any commercial or financial relationships that could be construed as a potential conflict of interest.

## Supplementary Material

Supplementary Figure 1 In IL-37 treated cells, there were 1,498 non-overlapping m6A peaks within 1,300 mRNAs and 180 non-overlapping m6A peaks within 175 lncRNAs in two biological replicates. Of these, 35 peaks within mRNAs were overlapped between the IL-37 treated and untreated cells (**Figure 3B**) and only one peak within lncRNAs was overlapped.

Supplementary Figure 2 m6A distribution of differentially methylated m6A sites for both IL-37 treated and untreated cells. **(A)** Chromosomal distribution of all differentially methylated m6A sites within mRNAs and lncRNAs; **(B)** Relative occupancy of differentially methylated m6A sites in chromosome; **(C)** The percentage of differentially methylated m6A sites; **(D)** Fold change of differentially methylated m6A sites.

Supplementary Figure 3 Results of the cellular component (CC) and molecular function (MF) analysis.
