# Supplementary figures and images for: IL-37 Confers Anti-Tumor Activity by Regulation of m6A Methylation
Source: Front Oncol. 2021 Jan 8;10:526866. doi: 10.3389/fonc.2020.526866 (PMC7821743; doi:10.3389/fonc.2020.526866)

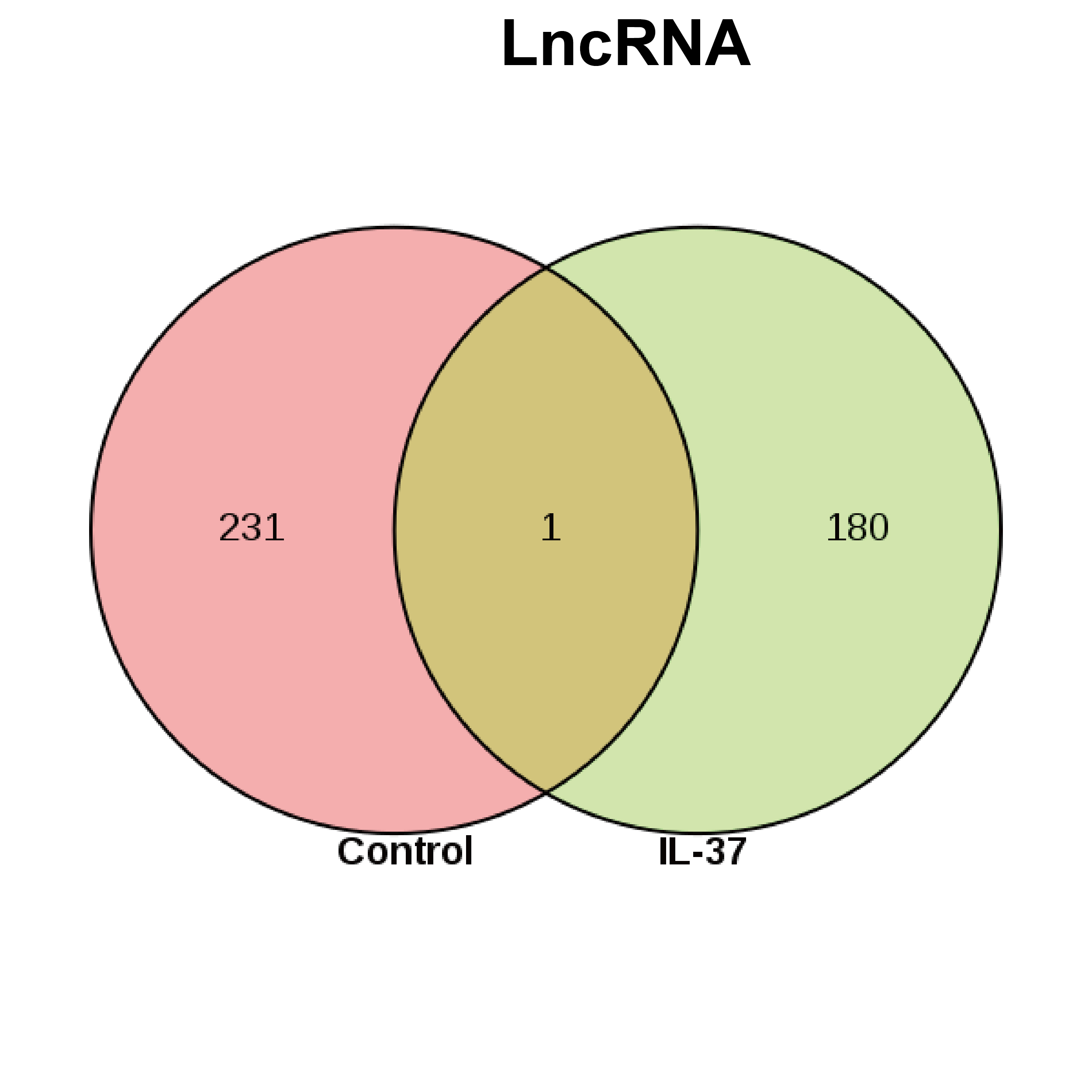

Supplement: Supplementary Figure 1 — In IL-37 treated cells, there were 1,498 non-overlapping m6A peaks within 1,300 mRNAs and 180 non-overlapping m6A peaks within 175 lncRNAs in two biological replicates. Of these, 35 peaks within mRNAs were overlapped between the IL-37 treated and untreated cells (Figure 3B) and only one peak within lncRNAs was overlapped. [file Image_1.jpeg]

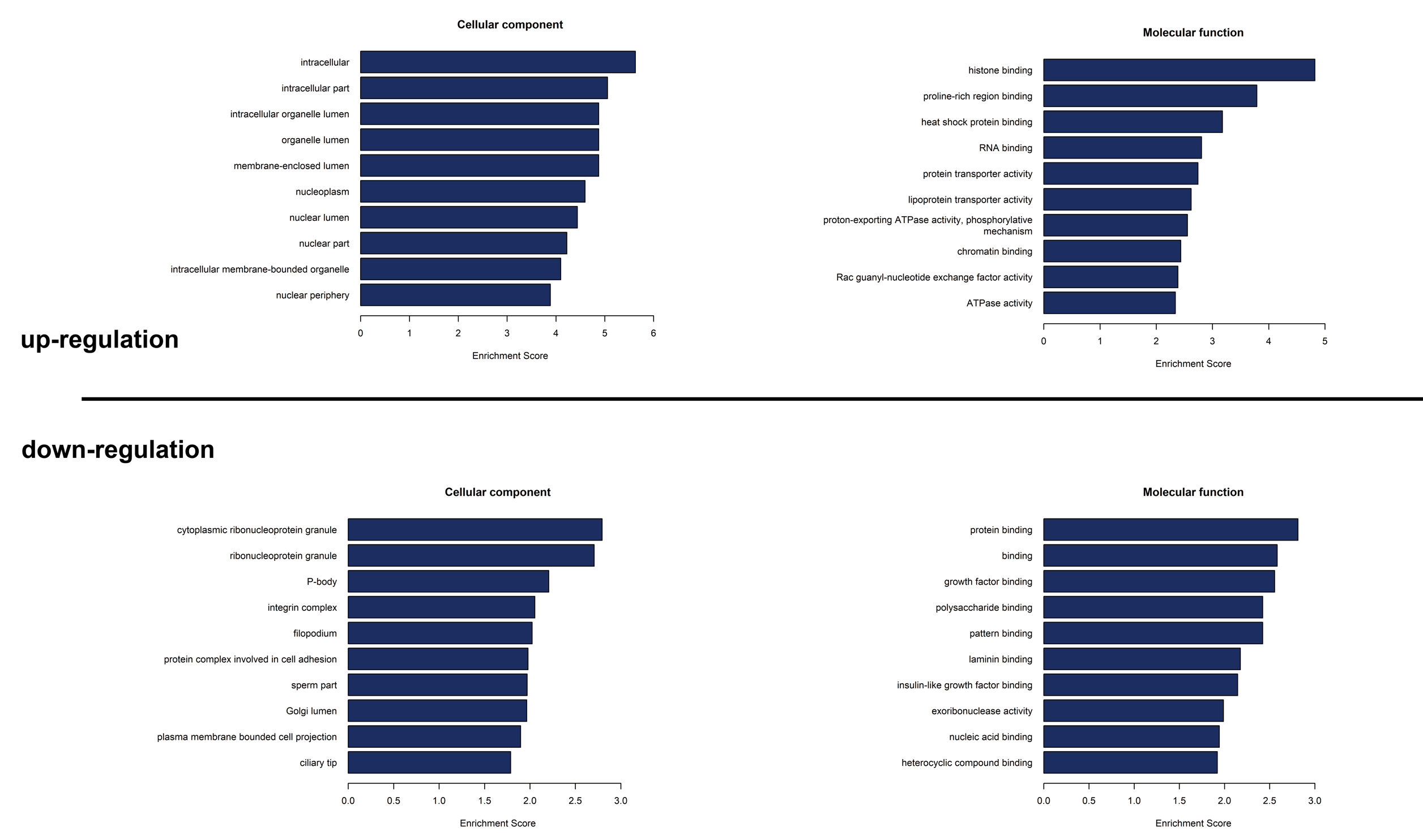

Supplement: Supplementary Figure 3 — Results of the cellular component (CC) and molecular function (MF) analysis. [file Image_3.jpeg]
